# Popularity versus quality in TikTok educational videos on congenital nasolacrimal duct obstruction

**DOI:** 10.3389/fmed.2026.1762313

**Published:** 2026-02-02

**Authors:** Manhui Zhu, Wenqun Xi, Lin Xu, Yuanyuan Tu, Jialuo Zhao, Yingxin Guo, Zhe Zhang, Zhentao Zhu, Ligang Jiang

**Affiliations:** 1 Department of Ophthalmology, Lixiang Eye Hospital of Soochow University, Suzhou, Jiangsu, China; 2 Department of Ophthalmology, Shenzhen Eye Hospital, Shenzhen Eye Medical Center, Southern Medical University, Shenzhen, China; 3 Department of Ophthalmology, Huai’an Clinical Medical College of Jiangsu University, Huai’an Hospital of Huai’an City, Huai’an, China; 4 Department of Ophthalmology, Quzhou Affiliated Hospital of Wenzhou Medical University, Quzhou People’s Hospital, Quzhou, Zhejiang, China

**Keywords:** congenital nasolacrimal duct obstruction, health education, health information quality, neonatal dacryocystitis, social media, TikTok

## Abstract

**Background:**

Congenital nasolacrimal duct obstruction is a common ocular condition in early infancy and may lead to neonatal dacryocystitis or severe infection if not treated promptly. Short-video platforms such as TikTok are used by young parents to obtain health information, but the quality of related videos remains unclear. This study evaluated the quality of TikTok videos on congenital nasolacrimal duct obstruction and its association with uploader type and user engagement.

**Methods:**

We conducted a cross-sectional review of TikTok videos retrieved with predefined keywords. We included 108 videos, classified by uploader type, and extracted characteristics, engagement metrics, and coverage of six content domains. Two attending ophthalmologists independently rated each video using DISCERN, the Global Quality Score, and the Patient Education Materials Assessment Tool for Audiovisual Materials. Group differences and Spearman correlations were analyzed.

**Results:**

Among 108 videos, 62 (57.4%) were uploaded by medical professionals and 18 (16.7%) by non-Profit organizations, with a median duration of 48.5 s and median numbers of likes, comments, favorites and shares of 68, 8, 13, and 24, respectively. Videos uploaded by non-Profit organizations and medical professionals achieved substantially higher DISCERN scores (about 59.22 and 50.00 vs. 37.50 and 19.40), Global Quality Scores (median 4.5 and 4.0 vs. 3.0 and 1.0), and Patient Education Materials Assessment Tool for Audiovisual Materials understandability (median 92.31 and 84.62% vs. 73.08 and 53.85%) and actionability (both 75.00% vs. 66.67 and 50.00%) than those uploaded by non-Medical individuals and for-profit organizations (all *P* < 0.001). Spearman analysis found no significant correlations between overall quality scores and engagement, but duration correlated weakly with some selected quality indicators (*r* = 0.17–0.21, *P* < 0.05).

**Conclusion:**

TikTok videos on congenital nasolacrimal duct obstruction show marked heterogeneity in content completeness and educational quality, largely determined by uploader type. Non-Profit organizations and medical professionals produce more reliable, understandable, and actionable videos, but these are not consistently more popular than lower-quality content. Clinicians and institutions should develop guideline-concordant short videos and direct parents toward trustworthy channels, while platforms consider mechanisms to highlight professionally verified pediatric eye-health information.

## 1 Introduction

Congenital nasolacrimal duct obstruction (CNLDO) is one of the most common congenital ocular conditions in early infancy and represents the underlying pathology in the majority of cases with neonatal epiphora (1, 2). It affects approximately 6–20% of infants in the first year of life and usually presents with persistent epiphora and mucous discharge due to distal nasolacrimal duct blockage at the valve of Hasner (3, 4). Most cases resolve spontaneously with maturation of the nasolacrimal drainage system (5–7). However, persistent or unrecognized CNLDO may predispose to recurrent infection, with neonatal dacryocystitis representing an acute infection of the lacrimal sac that can further progress to preseptal or orbital cellulitis and even systemic sepsis if not promptly treated (8–10). Current reviews and clinical guidelines emphasize early identification of symptoms by caregivers and primary-care providers, appropriate use of lacrimal sac massage and topical/systemic antibiotics (11, 12), and timely escalation to probing or surgery when obstruction persists (13–15).

In parallel, short-video platforms such as TikTok have rapidly become major sources of health information for young parents. While these platforms provide convenient, visually intuitive content, emerging evidence suggests substantial variability in the quality and reliability of medical videos (16, 17). Despite the high prevalence of CNLDO and the potential severity of neonatal dacryocystitis, the quality of TikTok videos addressing these conditions has not been systematically evaluated. Parents searching for “CNLDO” or “newborn eye discharge” may therefore be exposed to content that incompletely explains the natural course of CNLDO, underemphasizes warning signs of serious infection, or promotes non–evidence-based home remedies. This cross-sectional study aims to assess the quality, reliability and understandability of CNLDO–related videos on TikTok using validated evaluation tools, and to explore the association between video quality, uploader type and user engagement metrics.

## 2 Materials and methods

### 2.1 Platform and search strategy

**FIGURE 1:**
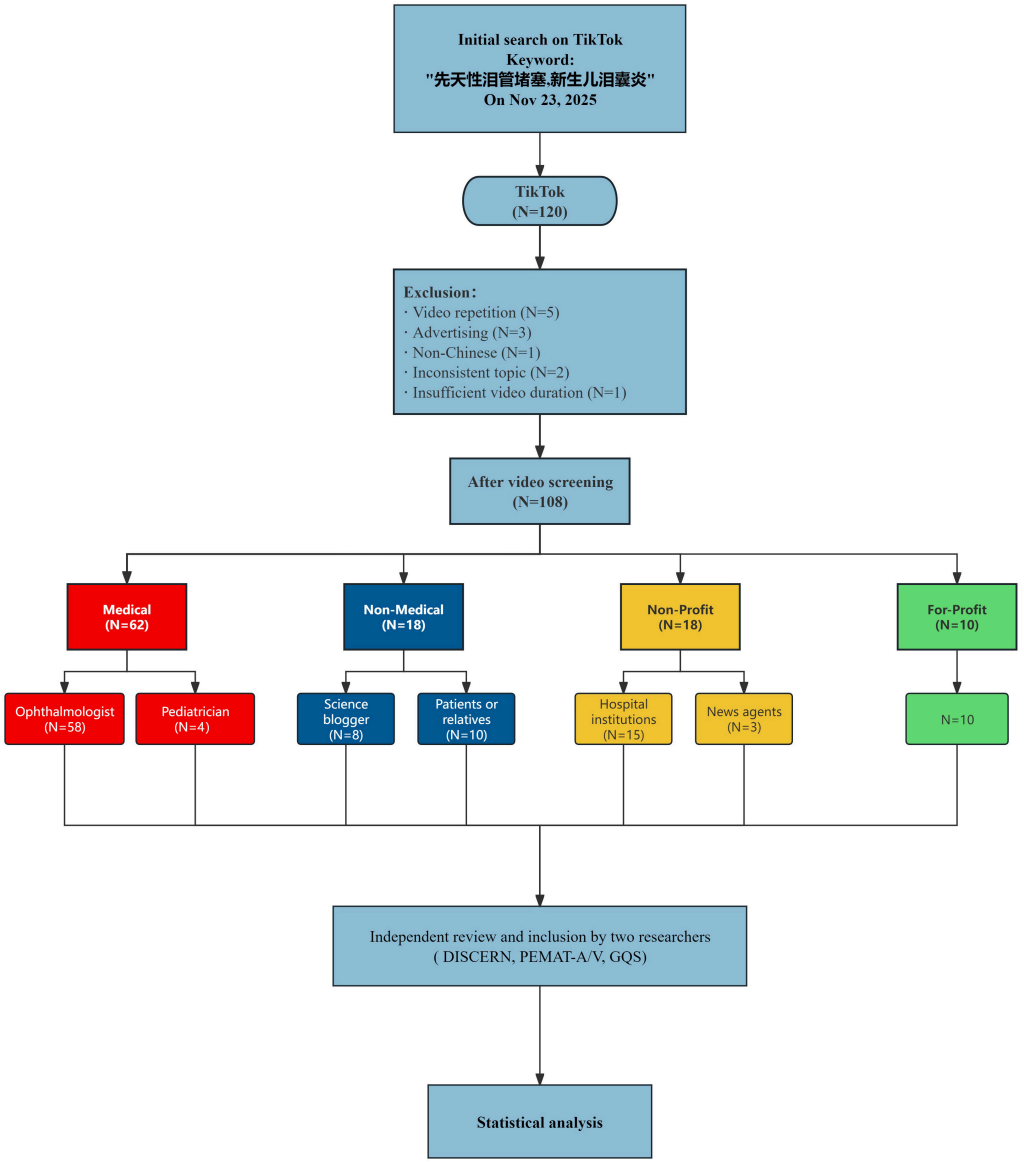
Flowchart of identification, screening, and classification of CNLDO-related TikTok videos.

TikTok (Chinese mainland version) was selected as the data source. A unified search was conducted on 23 November 2025 using the in-app search function. To approximate real-world search behavior of parents, we predefined combinations of Chinese keywords, including but not limited to “congenital nasolacrimal duct obstruction (先天性泪管堵塞)” and “neonatal dacryocystitis (新生儿泪囊炎)”. The platform’s default “comprehensive” ranking mode was applied. The top-ranked 120 videos were consecutively collected until a large number of duplicate or clearly irrelevant videos appeared, forming the initial candidate pool. The detailed screening process is shown in Figure 1.

### 2.2 Inclusion and exclusion criteria

Inclusion criteria were as follows: (1) videos primarily addressing issues related to neonatal or infant dacryocystitis or CNLDO, including any of the following content domains—definition, symptoms, risk factors, diagnostic examinations, treatment and management, and/or prognosis; (2) videos involving at least one of the above domains were eligible; (3) language in Mandarin Chinese or with Chinese subtitles to ensure assessors’ comprehension; and (4) publicly accessible complete videos.

Exclusion criteria were: (1) purely entertaining or humorous videos without any medical or health-related information; (2) videos mainly consisting of commercial advertisements or product promotion with no specific disease-related educational content; (3) videos unrelated to neonatal or infant ocular problems; (4) duplicate videos; (5) videos without sound and subtitles such that content could not be judged; and (6) videos that had been deleted, set to private, or could not be played normally by the platform. Two researchers independently screened all candidate videos according to these criteria. Disagreements were resolved through discussion or adjudication by a third researcher.

### 2.3 Video characteristics and uploader type

For each included video, data were extracted using a standardized data collection form. Video parameters: upload date; video duration (seconds); and engagement metrics including number of likes, comments, favorites, and shares, all recorded as displayed by the platform on the search date.

Uploader account characteristics: account name and verification status. Based on account verification information and profile descriptions, uploader type was categorized into four groups: (1) Medical (professional medical users, such as ophthalmologists or pediatricians); (2) Non-Medical (non-professional users, such as patients, parents, or science communicators); (3) Non-Profit organizations (for example, medical institutions or news organizations); and (4) For-Profit organizations (accounts primarily engaged in product marketing, such as postpartum care centers or maternal-and-child stores).

### 2.4 Video quality and credibility assessment tools

To ensure scientific rigor in the evaluation, we referred to relevant articles published in journals of the American Academy of Ophthalmology (AAO) as content evaluation standards (8, 18). Three validated instruments were used to comprehensively assess video quality and patient education value. All raters jointly reviewed the scoring manuals before formal assessment to ensure consistent understanding.

DISCERN instrument: The classic 16-item DISCERN tool was used to evaluate the reliability and quality of treatment-related information. Each item is scored from 1 to 5 (1 = “severely inadequate”; 5 = “excellent”), yielding a total score ranging from 16 to 80. Higher total scores indicate more reliable and higher-quality information on treatment options (16).

Global Quality Scale (GQS): The overall educational quality and viewing experience of each video were assessed using the 5-point GQS (1 = very poor quality, not helpful; 5 = excellent quality, highly recommended), integrating information accuracy, structural coherence, and practical usefulness (19).

Patient Education Materials Assessment Tool for Audiovisual Materials (PEMAT-A/V): The PEMAT-A/V was employed to evaluate understandability and actionability. Each item was rated as “yes,” “no,” or “not applicable.” Scores were converted to percentage values, with higher percentages indicating that patients would find the material easier to understand and act upon (20).

### 2.5 Rating procedure and inter-rater reliability

All included videos were independently rated by two attending ophthalmologists, each with approximately 5 years of clinical experience, who had received structured training in health education material assessment. During scoring, each rater watched the entire video in a quiet environment (including audio, subtitles, and on-screen images) and recorded item-by-item scores for each quality instrument.

To reduce potential bias, uploader identities were anonymized using coded identifiers, and raters did not view or record follower counts or other engagement metrics that might influence subjective judgments. The two raters completed their ratings independently at different times. Inter-rater reliability for each scoring tool was evaluated using the intraclass correlation coefficient (ICC). If the ICC for any instrument indicated poor agreement, the research team revisited the scoring criteria, discussed discrepant items, and refined the scoring instructions accordingly.

### 2.6 Statistical analysis

All data were entered into electronic spreadsheets by two researchers and cross-checked for accuracy. Statistical analyses were performed using IBM SPSS Statistics version 27.0 (IBM Corp., Armonk, NY, United States). Normality of continuous variables was assessed with the Shapiro–Wilk test. Normally distributed continuous variables were expressed as mean ± standard deviation, whereas non-normally distributed variables were presented as median and interquartile range [M (P25, P75)]. For comparisons among groups, one-way analysis of variance (ANOVA) or the Kruskal–Wallis test was used for continuous variables, depending on variance homogeneity and distribution. When overall differences were significant, post hoc pairwise comparisons were conducted using Bonferroni or Dunn–Bonferroni corrections as appropriate. Spearman or Pearson correlation analyses were used to examine associations between quality scores and user engagement metrics. All statistical tests were two-sided, and a *P* < 0.05 was considered statistically significant.

## 3 Results

### 3.1 Analysis of basic video characteristics

**FIGURE 2:**
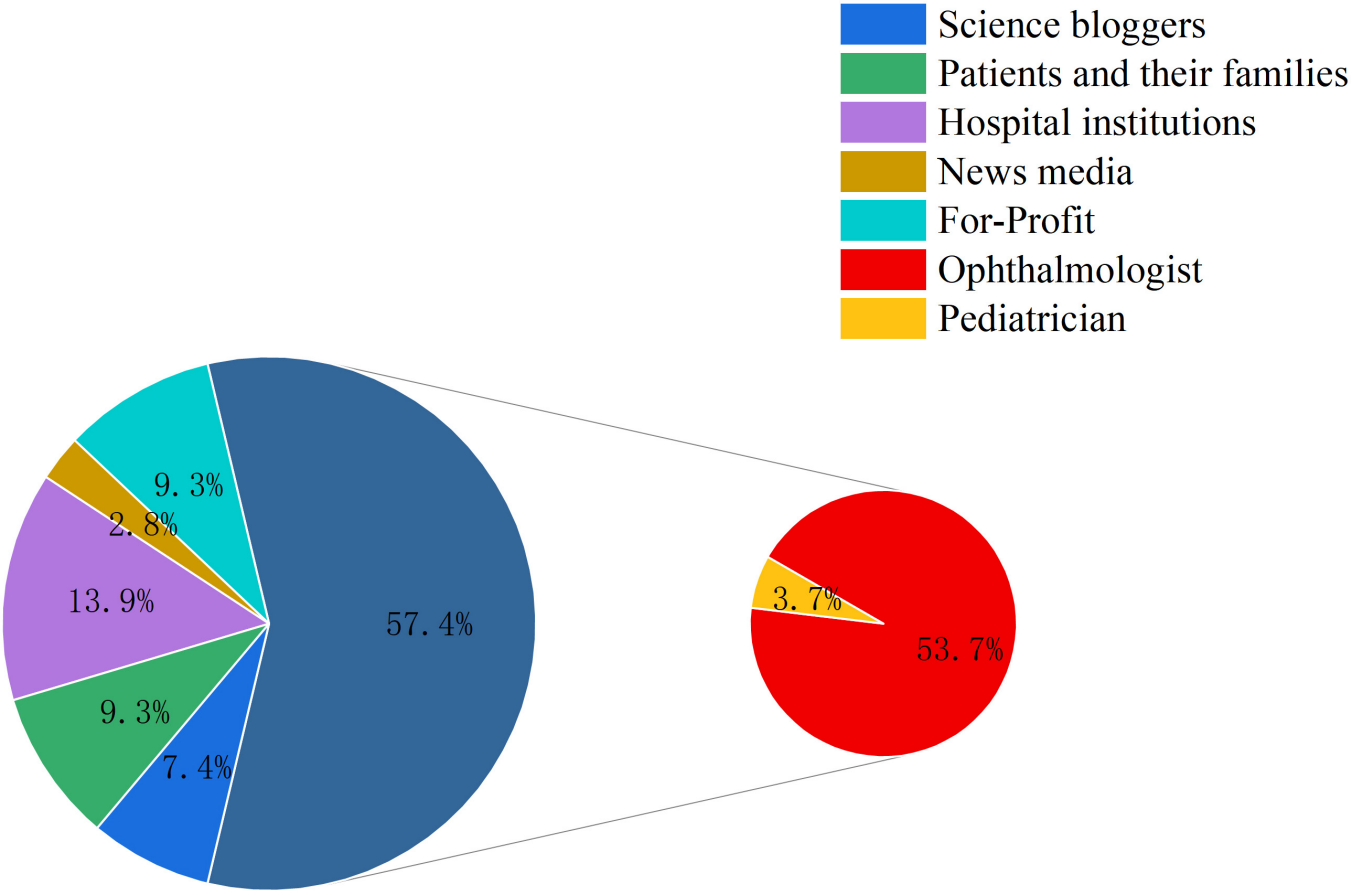
Distribution of uploader types for CNLDO-related TikTok videos.

A total of 108 TikTok videos related to congenital nasolacrimal duct obstruction were finally included. Most videos were uploaded by medical professionals: ophthalmologists uploaded 58 videos (53.7%) and pediatricians 4 videos (3.7%), for a total of 62 videos (57.4%). Non-Profit organizations (including hospitals and news media) uploaded 18 videos (16.7%), of which 15 (13.9%) were from hospitals and 3 (2.8%) from news media. Non-Medical individual users (science communicators and patients or family members) also uploaded 18 videos (16.7%), including 8 (7.4%) by science communicators and 10 (9.3%) by patients or caregivers. For-Profit organizations uploaded 10 videos (9.3%) (Table 1). Overall, the median numbers of likes, comments, favorites, and shares for the included videos were 68.0 (24.5, 228.0), 8.0 (1.25, 26.75), 12.5 (3.0, 55.25), and 23.5 (5.25, 102.75), respectively. The median video duration was 48.5 s (32.5, 72.75), indicating a moderate level of user engagement and dissemination for CNLDO-related content on the platform (Figure 2).

**TABLE 1:** Basic characteristics and engagement metrics of CNLDO-related TikTok videos by uploader type.

Video parameters	Individual users		Organizational users		Overall
	Medical (*n* = 62)	Non-Medical (*n* = 18)	Non-Profit (*n* = 18)	For-Profit (*n* = 10)	
Likes	52.00 (30.75, 115.50)	629.50 (167.50, 4491.50)	175.00 (13.50, 313.75)	7.60 ± 1.71	68.00 (24.50, 228.00)
Comments	5.50 (2.75, 15.25)	170.50 (27.50, 471.00)	9.00 (0.75, 66.50)	1.00 (0.00, 1.00)	8.00 (1.25, 26.75)
Favorites	11.00 (3.00, 34.75)	95.50 (19.75, 443.25)	17.00 (6.50, 51.25)	1.00 (0.00, 1.00)	12.50 (3.00, 55.25)
Shares	24.50 (7.75, 92.50)	154.50 (29.00, 285.25)	19.50 (9.00, 55.00)	0.00 (0.00, 1.00)	23.50 (5.25, 102.75)
Video duration	47.00 (35.00, 64.00)	76.17 ± 50.03	58.00 (49.50, 120.75)	22.50 ± 4.88	48.50 (32.50, 72.75)

**FIGURE 3:**
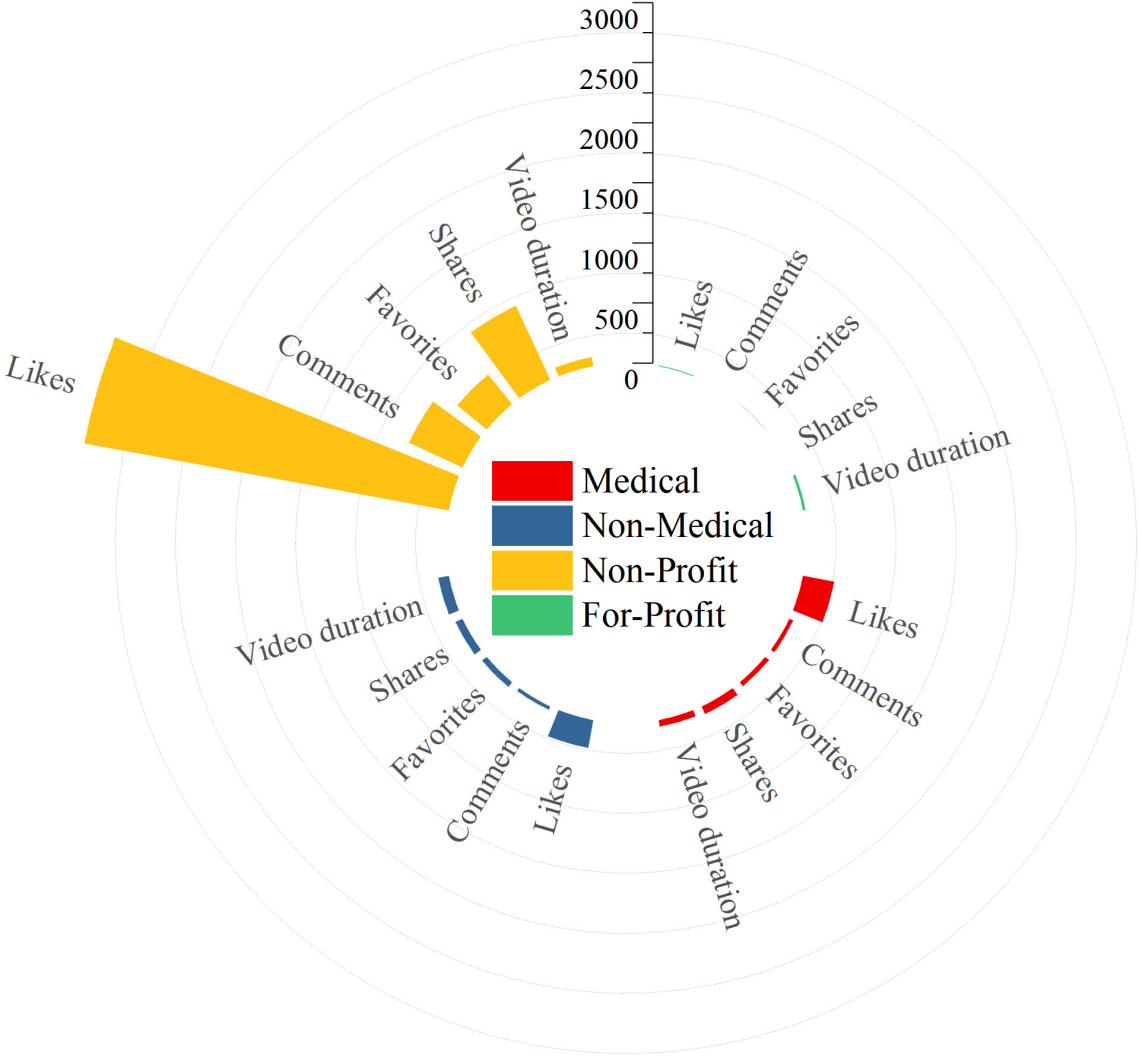
Engagement metrics of CNLDO-related TikTok videos by uploader type.

Marked differences in engagement metrics were observed across different uploader types. Videos uploaded by Non-Medical individual users received the highest engagement, with median numbers of likes, comments, favorites, and shares of 629.5 (167.5, 4491.5), 170.5 (27.5, 471.0), 95.5 (19.75, 443.25), and 154.5 (29.0, 285.25), respectively. Videos from Non-Profit organizations showed intermediate levels of engagement, with 175.0 (13.5, 313.75) likes and 19.5 (9.0, 55.0) shares. In comparison, videos from Medical individual users had slightly lower exposure and engagement than those from Non-Medical individual users, but still achieved a certain dissemination effect, with 52.0 (30.75, 115.50) likes and 24.5 (7.75, 92.50) shares. Videos uploaded by For-Profit organizations had the lowest engagement, with a mean of only about 7.60 ± 1.71 likes, median values of 1.0 for both comments and favorites, and a median of 0 shares, suggesting limited attention to their educational content. Regarding video duration, Non-Medical individual users produced the longest videos on average (76.17 ± 50.03 s), followed by Non-Profit organizations at 58.0 s (49.5, 120.75). Videos from Medical individual users had a moderate duration of 47.0 s (35.0, 64.0), whereas those from For-Profit organizations were the shortest (22.50 ± 4.88 s) (Table 1). Overall, videos uploaded by Medical individual users and Non-Profit organizations tended to cluster around 1 min in length, which is more consistent with the “short and concise” pattern favored for health education on short-video platforms (Figure 3).

### 3.2 Video content analysis

**FIGURE 4:**
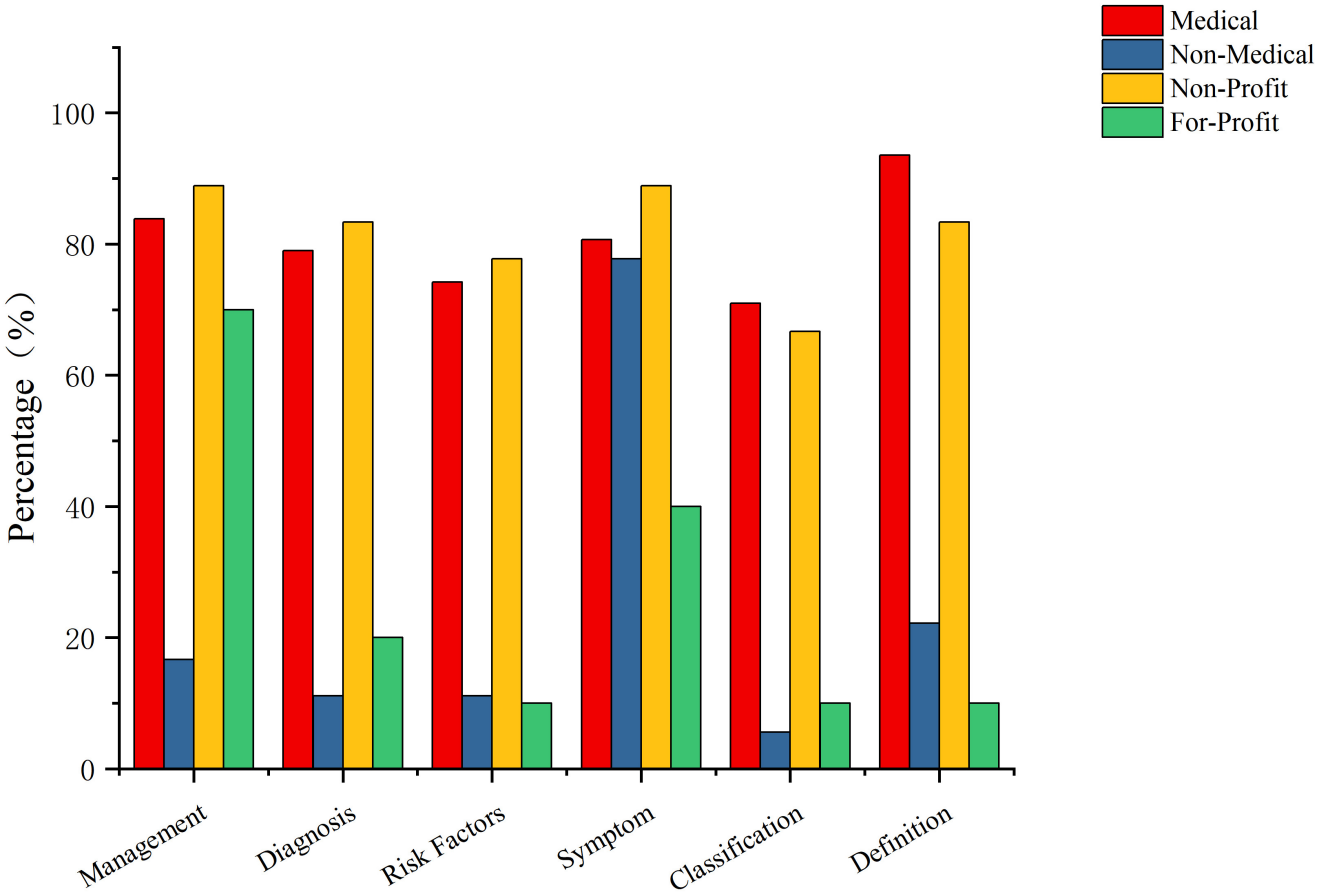
Content coverage of six core domains in CNLDO-related TikTok videos by uploader type.

The content of CNLDO-related videos was categorized into six core domains: disease definition, disease classification, clinical symptoms, risk factors, diagnostic examinations, and treatment and management. Among all 108 videos, clinical symptoms were the most frequently mentioned domain, covered in 84 videos (77.78%). Treatment and management and disease definition were tied for the second highest coverage, each appearing in 78 videos (72.22%). Diagnostic information was provided in 68 videos (62.96%), risk factors were mentioned in 63 videos (58.33%), and disease classification had the lowest coverage, being addressed in only 58 videos (53.70%). Overall, CNLDO-related TikTok videos tended to focus on “symptoms and management,” whereas systematic explanations of disease types and risk factors were relatively insufficient (Table 2; Figure 4).

**TABLE 2:** Content coverage of CNLDO-related TikTok videos across six core domains stratified by uploader type.

Core Domain	Medical (*n* = 62)	Non-Medical (*n* = 18)	Non-Profit (*n* = 18)	For-Profit (*n* = 10)
Definition	93.55%(58)	22.22%(4)	83.33%(15)	10.00%(1)
Classification	70.97%(44)	5.56%(1)	66.67%(12)	10.00%(1)
Symptoms	80.65%(50)	77.78%(14)	88.89%(16)	40.00%(4)
Risk Factors	74.19%(46)	11.11%(2)	77.78%(14)	10.00%(1)
Diagnosis	79.03%(49)	11.11%(2)	83.33%(15)	20.00%(2)
Management	83.87%(52)	16.67%(3)	88.89%(16)	70.00%(7)

As shown in Table 2, content coverage varied substantially across uploader types. Medical users (*n* = 62) performed best overall in all domains. In particular, 58 videos (93.55%) provided a relatively clear disease definition. High coverage was also observed for treatment and management (83.87%, 52/62), symptoms (80.65%, 50/62), diagnosis (79.03%, 49/62), and risk factors (74.19%, 46/62), while classification was mentioned in 70.97% of videos (44/62). Non-Profit organizations (*n* = 18, including hospitals and news media) also showed strong content integrity: coverage rates for symptoms and treatment and management both reached 88.89% (16/18), while definition and diagnosis were each present in 83.33% (15/18), risk factors in 77.78% (14/18), and classification in 66.67% (12/18), suggesting a certain advantage in systematic science popularization (Figure 4).

In contrast, videos from Non-Medical individual users (*n* = 18) and For-Profit organizations (*n* = 10) were clearly less comprehensive. Although Non-Medical individual users showed a relatively high coverage of symptoms (77.78%), coverage of definition (22.22%), treatment and management (16.67%), diagnosis and risk factors (both 11.11%), and classification (5.56%) was markedly inadequate. Videos from For-Profit organizations mainly focused on treatment and management (70.00%) and symptoms (40.00%), whereas coverage of definition, diagnosis, risk factors, and classification was only 10.00–20.00%. These findings indicate that, compared with Medical users and Non-Profit organizations, Non-Medical individual and commercial accounts have substantial gaps in delivering key disease information, which may lead to incomplete understanding or even misconceptions among parents (Figure 4).

Among the 108 included videos, the most common clinical pitfalls were lack of follow-up/re-evaluation advice (38/108, 35.19%) and lack of stepwise management and indications for procedures (37/108, 34.26%), followed by poor actionability/unclear caregiver instructions (32/108, 29.63%). Notably, these pitfalls were concentrated in the Non-Medical and For-Profit groups, with the highest counts observed for stepwise-management omissions (18 and 10 videos, respectively) and missing follow-up guidance (16 and 10 videos, respectively) (Table 3).

**TABLE 3:** Frequency of common clinical pitfalls and potentially harmful omissions identified in TikTok videos on CNLDO.

Clinical pitfall/omission (coded item)	Medical (*n* = 62)	Non-Medical (*n* = 18)	Non-Profit (*n* = 18)	For-Profit (*n* = 10)	Overall (*n* = 108)
Incorrect or unsafe lacrimal sac massage technique	0 (0.00%)	3 (2.78%)	0 (0.00%)	2 (1.85%)	5 (4.63%)
Overstating “watchful waiting/spontaneous resolution” without escalation criteria	1 (0.93%)	2 (1.85%)	0 (0.00%)	1 (0.93%)	4 (3.70%)
Omission of red-flag symptoms for urgent care	0 (0.00%)	3 (2.78%)	0 (0.00%)	6 (5.56%)	9 (8.33%)
Misleading antibiotic advice	0 (0.00%)	1 (0.93%)	0 (0.00%)	0 (0.00%)	1 (0.93%)
Misattribution to other causes without guidance	0 (0.00%)	2 (1.85%)	0 (0.00%)	2 (1.85%)	4 (3.70%)
Non-evidence-based home remedies or unsafe practices	0 (0.00%)	1 (0.93%)	0 (0.00%)	5 (4.63%)	6 (5.56%)
Lack of stepwise management and indications for procedures	8 (7.41%)	18 (16.67%)	1 (0.93%)	10 (9.26%)	37 (34.26%)
No follow-up/re-evaluation advice	11 (10.19%)	16 (14.81%)	1 (0.93%)	10 (9.26%)	38 (35.19%)
Poor actionability/unclear caregiver instructions	7 (6.48%)	15 (13.89%)	1 (0.93%)	9 (8.33%)	32 (29.63%)

### 3.3 Video quality analysis

**FIGURE 5:**
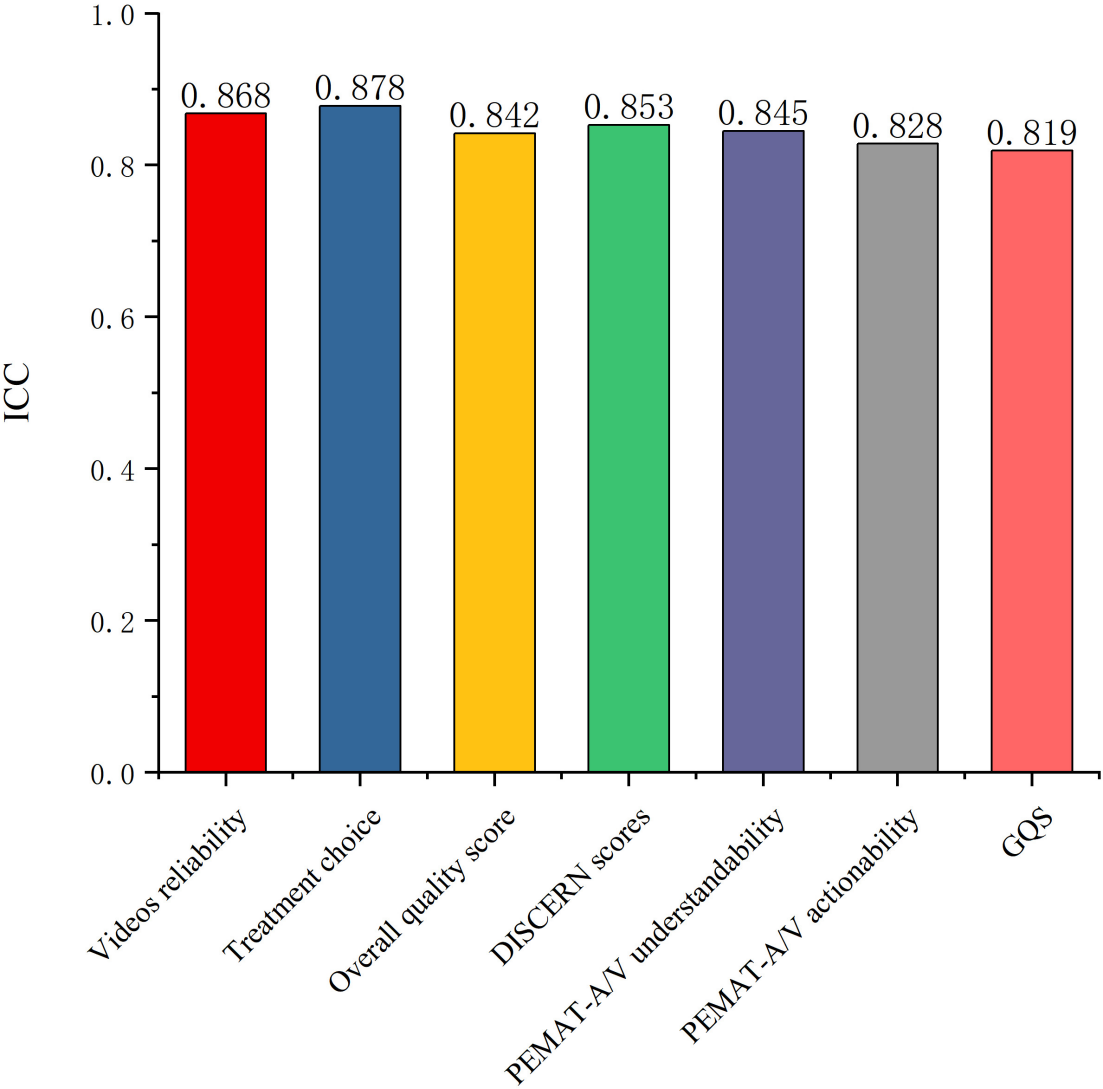
Inter-rater reliability of quality assessment for CNLDO-related TikTok videos.

For Videos reliability, Treatment choice, DISCERN scores, Overall quality score, Understandability, Actionability, and GQS, the intraclass correlation coefficients between the two raters were 0.868, 0.878, 0.842, 0.853, 0.845, 0.828, and 0.819, respectively (all *P* < 0.001) (Figure 5). Video quality among different uploader types was then compared based on the DISCERN instrument, PEMAT-A/V, and GQS (Table 4).

**TABLE 4:** Comparison of video quality scores among different uploader types based on DISCERN, PEMAT-A/V, and GQS.

Variables	Individual users		Organizational users		*H*-value	*P*-value
	Medical (*n* = 62)	Non-Medical (*n* = 18)	Non-Profit (*n* = 18)	For-Profit (*n* = 10)		
Videos reliability	27.00 (27.00, 28.00)	21.00 (17.00, 23.00)	32.39 ± 1.85	8.60 ± 1.67	88.15	**<0.001**
Treatment choice	19.00 (18.00, 21.00)	14.50 (13.00, 17.00)	23.00 ± 2.11	9.40 ± 1.27	73.19	**<0.001**
Overall quality score	3.00 (2.00, 4.00)	2.00 (2.00, 3.00)	4.00 (3.00, 4.00)	1.00 (1.00, 2.00)	38.17	**<0.001**
DISCERN scores	50.00 (49.00, 51.00)	37.50 (32.00, 42.00)	59.22 ± 2.51	19.40 ± 1.90	85.92	**<0.001**
Understandability	84.62 (76.92, 84.62)	73.08 (59.62, 76.92)	92.31 (84.62, 92.31)	53.85 (53.85, 55.77)	48.59	**<0.001**
Actionability	75.00 (66.67, 75.00)	66.67 (50.00, 66.67)	75.00 (75.00, 100.00)	50.00 (50.00, 50.00)	48.49	**<0.001**
GQS	4.00 (4.00, 4.00)	3.00 (2.00, 3.00)	4.50 (4.00, 5.00)	1.00 (1.00, 2.00)	65.98	**<0.001**

Values in bold indicate statistical significance (*P* < 0.05).

**FIGURE 6:**
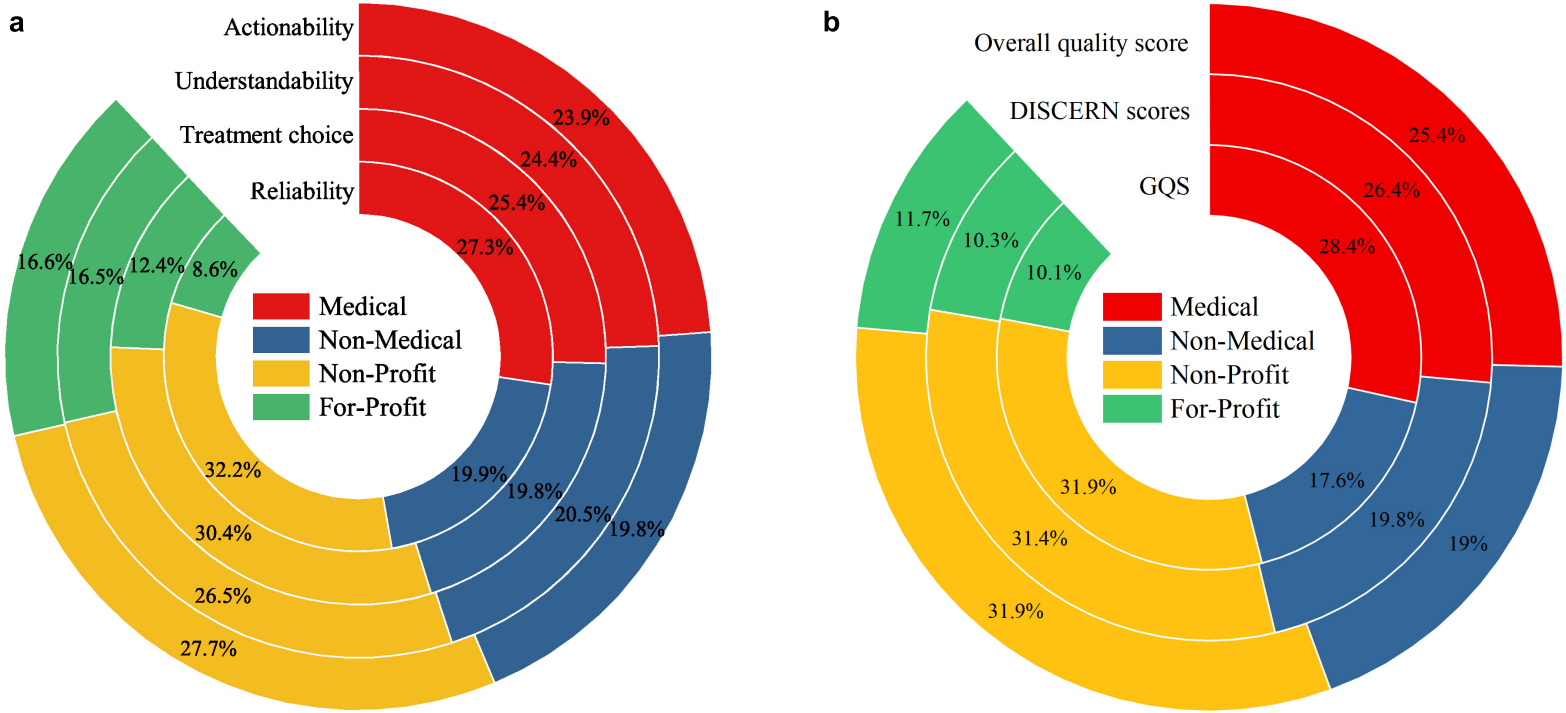
Distribution of quality indicators of CNLDO-related TikTok videos by uploader type.**(a)** Reliability, treatment choice, understandability and actionability; **(b)** Overall quality score, DISCERN scores and GQS.

Overall, videos from Non-Profit organizations had the highest quality indicators: the Videos reliability score was 32.39 ± 1.85, the Treatment choice score was 23.00 ± 2.11, the median Overall quality score was 4.00 (3.00, 4.00), and the DISCERN scores were 59.22 ± 2.51. Medical individual users ranked second, with a median Videos reliability score of 27.00 (27.00, 28.00), a median Treatment choice score of 19.00 (18.00, 21.00), a median Overall quality score of 3.00 (2.00, 4.00), and a median DISCERN scores of 50.00 (49.00, 51.00). Non-Medical individual users showed clearly lower scores in all domains, whereas For-Profit organizations had the lowest scores: their Videos reliability and Treatment choice scores were only 8.60 ± 1.67 and 9.40 ± 1.27, respectively, with a DISCERN scores of 19.40 ± 1.90 and a median Overall quality score of 1.00 (1.00, 2.00). Kruskal–Wallis tests indicated statistically significant differences among the four uploader types in Videos reliability, Treatment choice, Overall quality score, and DISCERN scores (*H* = 38.17–88.15, all *P* < 0.001) (Figure 6a).

In terms of Understandability and Actionability, Non-Profit organizations again performed best, with median scores of 92.31 (84.62, 92.31) and 75.00 (75.00, 100.00), both clearly above the 70% threshold commonly considered acceptable. Medical individual users also achieved favorable levels, with median Understandability and Actionability scores of 84.62 (76.92, 84.62) and 75.00 (66.67, 75.00), respectively. Although Non-Medical individual users showed a moderate Understandability score of 73.08 (59.62, 76.92), their Actionability score was only 66.67 (50.00, 66.67), close to or slightly below the acceptable threshold. For-Profit organizations had the lowest scores for both indicators, with Understandability of 53.85 (53.85, 55.77) and Actionability of 50.00 (50.00, 50.00). Regarding GQS, Non-Profit organizations and Medical individual users achieved median scores of 4.50 (4.00, 5.00) and 4.00 (4.00, 4.00), respectively, whereas Non-Medical individual users and For-Profit organizations scored only 3.00 (2.00, 3.00) and 1.00 (1.00, 2.00). Differences in Understandability, Actionability, and GQS among uploader types were all statistically significant (*H* = 48.49–65.98, all *P* < 0.001) (Figure 6b).

**FIGURE 7:**
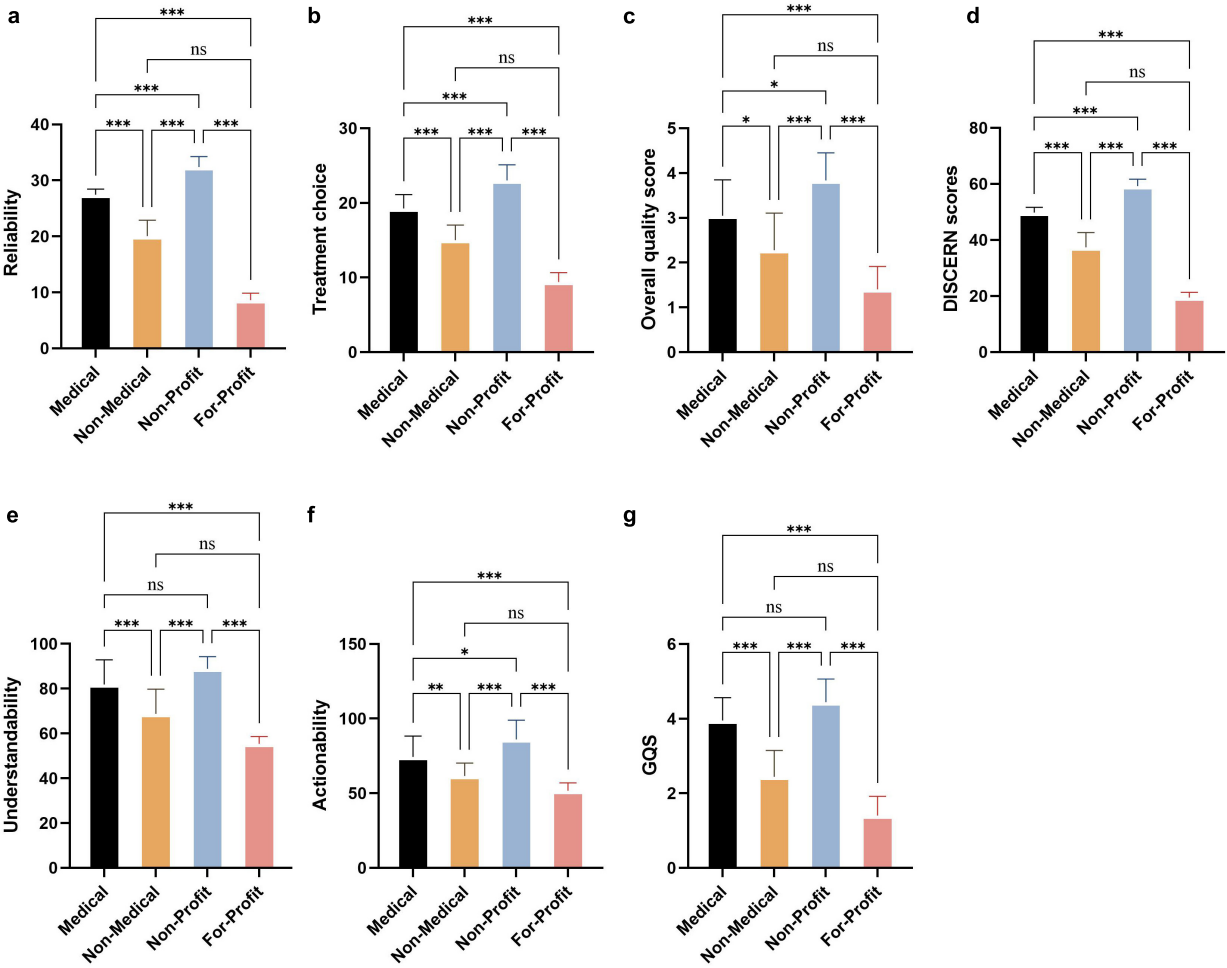
Comparison of quality indicators of CNLDO-related TikTok videos among different uploader types. **(a)** Reliability; **(b)** treatment choice; **(c)** overall quality score; **(d)** DISCERN scores; **(e)** understandability; **(f)** actionability; **(g)** GQS. *P < 0.05, **P < 0.01, ***P < 0.001, and ns *P*≥0.05.

Post hoc pairwise comparisons (Tables 5, 6) further demonstrated that videos uploaded by Non-Profit organizations were significantly superior to those from Non-Medical individual users and For-Profit organizations in Videos reliability, Treatment choice, Overall quality score, DISCERN scores, Understandability, Actionability, and GQS (multiple comparisons, *P* < 0.001 for many contrasts). Compared with Medical individual users, Non-Profit organizations also showed advantages in Videos reliability, Treatment choice, and DISCERN scores. Medical individual users had significantly higher Overall quality, Understandability, and GQS than Non-Medical individual users and For-Profit organizations (multiple comparisons, *P* < 0.05). In contrast, differences between Non-Medical individual users and For-Profit organizations were not significant for several indicators, including Videos reliability, Treatment choice, Understandability, Actionability, and GQS (*P* > 0.05). Taken together, these findings suggest that the quality of CNLDO-related videos on TikTok is highly dependent on uploader type: information provided by Non-Profit organizations and Medical professionals is relatively more reliable, easier to understand, and more actionable, whereas videos from commercial accounts and some Non-Medical individuals are generally of low quality (Figure 7).

**TABLE 5:** Pairwise comparisons of DISCERN-based quality indicators between different uploader types.

Comparison group	Videos reliability	Treatment choice	Overall quality score	DISCERN scores
Medical vs. Non-Medical	<0.001	0.390	0.026	<0.001
Medical vs. Non-Profit	<0.001	<0.001	0.003	<0.001
Medical vs. For-Profit	<0.001	<0.001	<0.001	<0.001
Non-Medical vs. Non-Profit	<0.001	<0.001	<0.001	<0.001
Non-Medical vs. For-Profit	0.244	0.142	0.025	0.246
Non-Profit vs. For-Profit	<0.001	<0.001	<0.001	<0.001

**TABLE 6:** Pairwise comparisons of understandability, actionability, and GQS between different uploader types.

Comparison group	Understandability	Actionability	GQS
Medical vs. Non-Medical	<0.001	0.002	<0.001
Medical vs. Non-Profit	0.090	0.020	0.220
Medical vs. For-Profit	<0.001	<0.001	<0.001
Non-Medical vs. Non-Profit	<0.001	<0.001	<0.001
Non-Medical vs. For-Profit	0.630	0.930	0.999
Non-Profit vs. For-Profit	<0.001	0.003	<0.001

### 3.4 Correlation analysis

**FIGURE 8:**
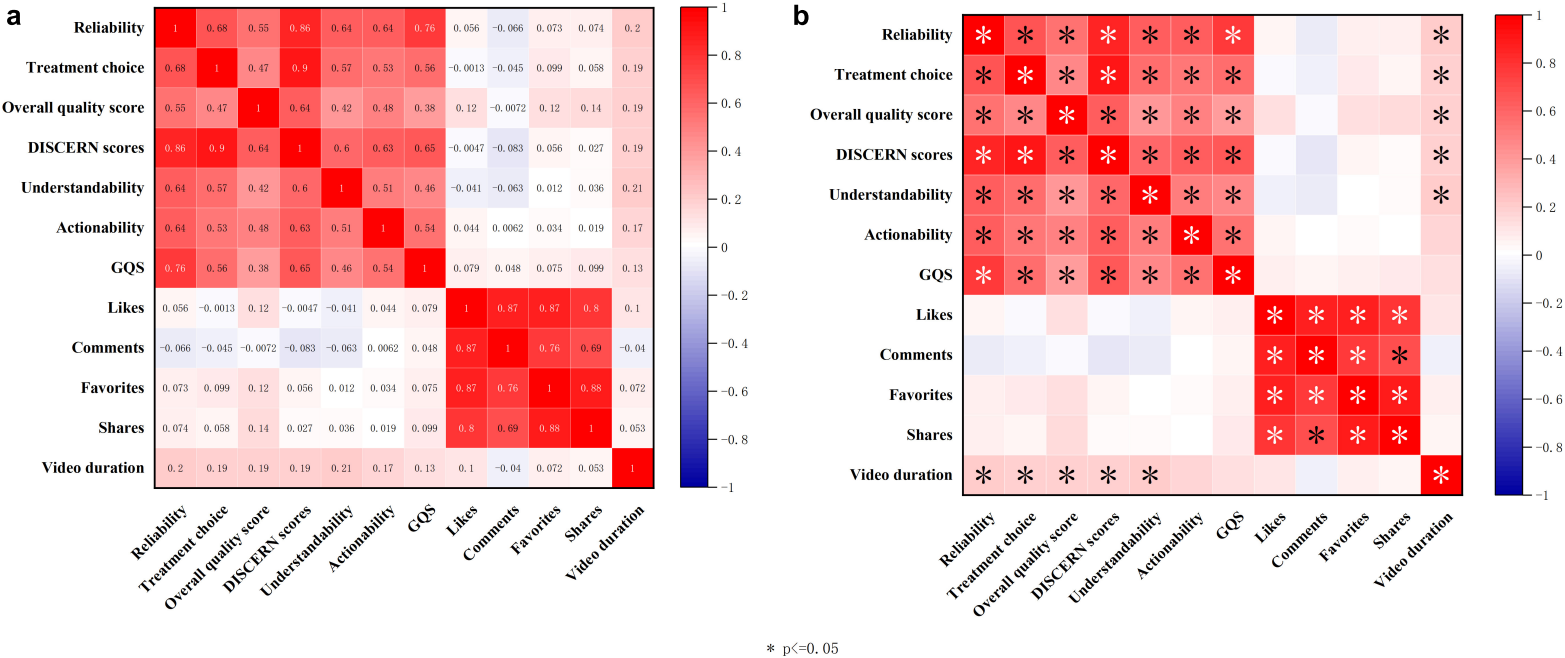
Correlations between quality indicators and engagement metrics of CNLDO-related TikTok videos. **(a)** Spearman correlation matrix with significance marks (asterisks indicate *P* < 0.05). **(b)** Heatmap of Spearman correlation coefficients for quality and engagement indicators.

Spearman correlation analysis was used to explore associations between video quality indicators and dissemination metrics. As shown in Figure 8, within the set of quality-related indicators, Videos reliability, Treatment choice, Overall quality score, DISCERN scores, Understandability, Actionability, and GQS were all moderately to highly positively correlated with one another (*r* ≈ 0.42–0.90, all *P* < 0.05). Among these, Videos reliability showed the strongest correlation with DISCERN scores (*r* ≈ 0.86), and was also highly correlated with GQS (*r* ≈ 0.76), indicating that more reliable videos tend to have higher levels of evidence and better overall subjective quality. The correlation coefficient between Treatment choice and DISCERN scores was close to 0.90, suggesting strong consistency between the completeness of treatment information and the overall evidence-based quality. Understandability and Actionability were also significantly and positively correlated (*r* ≈ 0.51), implying that videos that are easier to understand are more likely to provide parents with actionable recommendations.

In contrast, correlations between quality indicators and engagement metrics (likes, comments, favorites, and shares) were generally weak, with most correlation coefficients close to 0 and not statistically significant (*r* ≤ 0.12, *P* > 0.05). Only video duration showed a low positive correlation with some quality indicators (for example, *r* ≈ 0.17–0.21 with Videos reliability and DISCERN scores, *P* < 0.05). This suggests that on TikTok, engagement metrics do not have a simple linear relationship with the health education quality of videos. On the other hand, very strong correlations were observed among likes, comments, favorites, and shares (*r* ≈ 0.69–0.88, all *P* < 0.001), indicating that these engagement metrics mainly reflect a common dimension of “popularity” rather than professional quality per se. Overall, the professional quality of CNLDO-related videos appears to be determined primarily by the content itself and is only weakly associated with platform engagement levels (Figure 8).

## 4 Discussion

To our knowledge, this is the first study to systematically evaluate the content and quality of TikTok videos on congenital nasolacrimal duct obstruction and to compare these parameters across different uploader types. Overall, three major findings emerged. First, the number of CNLDO-related videos on the platform was limited, and most were uploaded by ophthalmologists, pediatricians, or medical institutions; however, information on symptoms and management was far more common than basic knowledge such as disease definition, classification, and risk factors, indicating an unbalanced content structure. S, video quality was highly dependent on uploader type: videos from Non-Profit organizations and Medical professionals clearly outperformed those from Non-Medical individuals and For-Profit organizations in terms of reliability, treatment information, Understandability, Actionability, and GQS. Third, strong correlations were observed among the various quality indicators themselves, whereas correlations between quality indices and engagement metrics (likes, comments, favorites, and shares) were weak, suggesting that popularity does not equate to health information quality.

For young parents who rely on short-video platforms for health information (21), whether videos convey key diagnostic and management checkpoints accurately is directly related to the risk of delayed consultation or inappropriate self-management (22). In this study, although more than 70% of videos mentioned disease symptoms and management strategies, only about half provided a systematic description of disease classification and risk factors. Some videos confused neonatal dacryocystitis with other causes of neonatal ocular discharge, or downplayed “red-flag” signs such as acute dacryocystitis and systemic infection. These findings contrast sharply with the integrated educational goals of CNLDO clinical guidelines, which emphasize the natural history, warning signs, and appropriate timing of intervention (23). If parents mainly encounter short videos that stress messages such as “most cases resolve spontaneously” or “just massage more and it will be fine” without adequately explaining high-risk features and clear indications for medical evaluation, recognition of serious complications may be delayed, imposing unnecessary burdens on affected children and their families (24, 25).

Consistent with TikTok-based studies of cardiovascular, endocrine, and other ocular diseases (26–31), we also observed pronounced differences across uploader types. Non-Profit organizations (including hospitals and news media) achieved the highest scores for Videos reliability, treatment information, Overall quality score, DISCERN scores, and the PEMAT-A/V domains of Understandability and Actionability. Their quality levels were comparable to those reported for high-quality short videos on coronary heart disease, thyroid nodules, cataract, and dry eye (26, 29–31). Medical individual users performed slightly less well than institutional accounts, particularly in the rigor of treatment descriptions and the degree of structured presentation, which may reflect constraints on personal creators in terms of time and resources. In contrast, videos from Non-Medical individuals and For-Profit organizations frequently showed information gaps or excessive simplification. In this study, both uploader types scored significantly lower than Medical users and Non-Profit organizations on DISCERN, PEMAT-A/V, and GQS, and some videos even recommended folk remedies or commercial services lacking evidence support. This pattern aligns closely with findings from short-video analyses of mitral valve regurgitation, coronary heart disease, and myopia (32–34), which indicate that commercial accounts tend to highlight products or services while neglecting comprehensive disease background and risk communication. For clinicians and public health practitioners, these results underscore the need to actively guide parents toward content produced by hospitals, professional societies, or verified expert accounts, while remaining alert to potential information bias from marketing-oriented channels (35, 36).

It is noteworthy that we found moderate-to-strong positive correlations among the video quality indicators, but no significant correlations between quality and engagement metrics such as likes, comments, favorites, and shares; only video duration showed a weak positive association with several quality indices. Similar phenomena have been reported in multiple studies on YouTube and TikTok (37–39), where high-quality health information does not necessarily achieve more likes or views, whereas “eye-catching” titles, dramatic visuals, or emotionally charged narratives are more likely to drive user behavior. Thus, treating engagement data as a simple proxy for “high-quality content” may systematically underestimate truly evidence-based educational materials. From an algorithmic perspective, recommender systems tend to favor content with high engagement and long viewing time, which may inadvertently amplify the spread of low-quality or even misleading medical information (40, 41). This suggests that medical professionals creating short videos should not only ensure accuracy and clear structure but also draw on strategies from digital health communication and behavioral science (42, 43), such as narrative storytelling, visualized presentation, and action-oriented conclusions, to improve viewing and sharing without compromising scientific rigor (44, 45).

Our findings support the incorporation of standardized evaluation tools, such as DISCERN and PEMAT-A/V, into short-video health education to guide content creation and quality control (46). By checking scripts against these instruments item by item before filming, creators can systematically verify whether they have adequately explained disease definition, risk factors, available treatment options, benefits and risks, and specific actions that parents can take. In addition, medical institutions could establish “official recommended accounts” and themed playlists to aggregate high-quality videos that have undergone peer or multidisciplinary review, thereby reducing parents’ screening burden in an information-saturated environment. For platform governance, it may be feasible—without making explicit medical judgments—to introduce structural quality signals, such as whether risk warnings are listed, whether clear consultation suggestions are provided, and whether sources are cited, so that recommendation algorithms can assign more weight to potentially high-quality medical content (47, 48). At the same time, further enhancing the visibility of professional identity verification and nudging users toward following credentialed physicians or medical institutions may help improve the overall quality of health information that parents encounter (49).

This study has several limitations. First, it was a cross-sectional analysis conducted at a single time point. The number, ranking, and engagement metrics of TikTok videos change dynamically; therefore, our findings represent only a “snapshot” of the search date and cannot capture temporal trends in content evolution. Second, we examined only videos on the TikTok platform and did not include other languages or platforms; thus, the generalizability of our conclusions to different cultural and platform contexts may be limited. Third, although the “Non-Profit” uploader category may comprise heterogeneous entities (e.g., hospital-based institutional accounts vs. news media accounts), the news media subgroup was small in our sample. Further stratification would likely reduce statistical power and yield unstable estimates; Fourth, although we used validated tools such as DISCERN and PEMAT-A/V and adopted a dual-rater approach to enhance consistency, assessment of video quality inevitably retains some degree of subjectivity. Fifth, the present study primarily focused on the completeness and presentation of information and did not verify each statement against the latest clinical guidelines on an item-by-item basis. Future work could perform more fine-grained quantitative analyses of content accuracy and transparency of risk communication. Sixth, we did not analyze comment sections or user interaction content, which may further influence parental perceptions and behaviors. These limitations highlight the need for future multicenter studies conducted at multiple time points, across different platforms, and with larger samples, ideally incorporating behavioral outcomes, to more comprehensively evaluate the real-world impact of short-video health information on CNLDO management.

## 5 Conclusion

This study systematically evaluated the content and quality of TikTok videos related to congenital nasolacrimal duct obstruction. Video quality was closely associated with uploader type: videos posted by Non-Profit organizations and Medical professionals demonstrated markedly higher reliability, treatment information quality, overall quality, and better Understandability and Actionability than those from Non-Medical individuals and For-Profit accounts, whereas correlations with engagement metrics were weak—“popular” did not necessarily mean “high-quality” (50). In the future, clinicians and public health professionals should be encouraged to actively participate in creating authoritative educational content, to guide parents toward information from trustworthy accounts, and to advocate for further optimization of platform mechanisms for professional verification and quality signaling (51).

## Data Availability

The original contributions presented in the study are included in the article/supplementary material, further inquiries can be directed to the corresponding authors.
